# Supplementary material for: Origin of conformational dynamics in a globular protein
Source: Commun Biol. 2019 Nov 26;2:433. doi: 10.1038/s42003-019-0681-2 (PMC6879633; doi:10.1038/s42003-019-0681-2)
Supplement: Supplementary file 2 — Reporting Summary [file 42003_2019_681_MOESM2_ESM.pdf]

nature research

Corresponding author(s): Roberto A. Chica

Last updated by author(s): Aug 23, 2019

Reporting Summary

Nature Research wishes to improve the reproducibility of the work that we publish. This form provides structure for consistency and transparency in reporting. For further information on Nature Research policies, see Authors & Referees and the Editorial Policy Checklist.

Statistics

For all statistical analyses, confirm that the following items are present in the figure legend, table legend, main text, or Methods section.

n/a	Confirmed	
☐	☒	The exact sample size (*n*) for each experimental group/condition, given as a discrete number and unit of measurement
☐	☒	A statement on whether measurements were taken from distinct samples or whether the same sample was measured repeatedly
☒	☐	The statistical test(s) used AND whether they are one- or two-sided<br>*Only common tests should be described solely by name; describe more complex techniques in the Methods section.*
☒	☐	A description of all covariates tested
☐	☒	A description of any assumptions or corrections, such as tests of normality and adjustment for multiple comparisons
☐	☒	A full description of the statistical parameters including central tendency (e.g. means) or other basic estimates (e.g. regression coefficient) AND variation (e.g. standard deviation) or associated estimates of uncertainty (e.g. confidence intervals)
☒	☐	For null hypothesis testing, the test statistic (e.g. *F*, *t*, *r*) with confidence intervals, effect sizes, degrees of freedom and *P* value noted<br>*Give P values as exact values whenever suitable.*
☒	☐	For Bayesian analysis, information on the choice of priors and Markov chain Monte Carlo settings
☒	☐	For hierarchical and complex designs, identification of the appropriate level for tests and full reporting of outcomes
☒	☐	Estimates of effect sizes (e.g. Cohen's *d*, Pearson's *r*), indicating how they were calculated

Our web collection on statistics for biologists contains articles on many of the points above.

Software and code

Policy information about availability of computer code

Data collection	Molecular dynamics simulations were performed using in-house code written with the OpenMM API (version 7, http://openmm.org/).<br>NMR data acquisition was performed using Bruker Topspin version 3.5.
Data analysis	Data analysis for molecular dynamics simulations, circular dichroism measurement, and melting curves was performed using in-house code, VMD, and Microsoft Excel.<br>NMR data processing and analysis was performed using Bruker Topspin version 3.5 and NMRViewJ.<br>The NMR structure of D3-F34A was solved using CYANA version 2.1 with dihedral angle restraints calculated using TALOS+.<br>Protein structures were visualized using PyMol version 1.3 (academic use).

For manuscripts utilizing custom algorithms or software that are central to the research but not yet described in published literature, software must be made available to editors/reviewers. We strongly encourage code deposition in a community repository (e.g. GitHub). See the Nature Research guidelines for submitting code & software for further information.

Data

Policy information about availability of data

All manuscripts must include a data availability statement. This statement should provide the following information, where applicable:

- Accession codes, unique identifiers, or web links for publicly available datasets
- A list of figures that have associated raw data
- A description of any restrictions on data availability

The datasets generated during and/or analysed during the current study are available from the corresponding author on reasonable request.

Structure coordinates for D3-F34A have been deposited in the Protein Data Bank with the accession code 6NJF. NMR data for D3-F34A has been deposited in the Biological Magnetic Resonance Data Bank with the accession code 30532.

Field-specific reporting

Please select the one below that is the best fit for your research. If you are not sure, read the appropriate sections before making your selection.

☒ Life sciences ☐ Behavioural & social sciences ☐ Ecological, evolutionary & environmental sciences

For a reference copy of the document with all sections, see nature.com/documents/nr-reporting-summary-flat.pdf

Life sciences study design

All studies must disclose on these points even when the disclosure is negative.

Sample size	No sample size calculations were performed. Experiments were repeated in duplicate or triplicate where possible.
Data exclusions	No data was excluded from analyses.
Replication	Experiments were repeated in duplicate or triplicate where feasible. All replications were successful.
Randomization	This is not relevant to our study as we studied a purified protein system in absence of any host organism.
Blinding	This is not relevant to our study as we studied a purified protein system in absence of any host organism.

Reporting for specific materials, systems and methods

We require information from authors about some types of materials, experimental systems and methods used in many studies. Here, indicate whether each material, system or method listed is relevant to your study. If you are not sure if a list item applies to your research, read the appropriate section before selecting a response.

Materials & experimental systems

n/a	Involved in the study	
☒	☐	Antibodies
☒	☐	Eukaryotic cell lines
☒	☐	Palaeontology
☒	☐	Animals and other organisms
☒	☐	Human research participants
☒	☐	Clinical data

Methods

n/a	Involved in the study	
☒	☐	ChIP-seq
☒	☐	Flow cytometry
☒	☐	MRI-based neuroimaging
